# Pharmacodynamic Evaluation of Shenfu Injection in Rats With Ischemic Heart Failure and Its Effect on Small Molecules Using Matrix-Assisted Laser Desorption/Ionization–Mass Spectrometry Imaging

**DOI:** 10.3389/fphar.2019.01424

**Published:** 2019-11-26

**Authors:** Hao Wu, Zhenfeng Dai, Xi Liu, Ming Lin, Zeyu Gao, Fang Tian, Xin Zhao, Yi Sun, Xiaoping Pu

**Affiliations:** ^1^National Key Research Laboratory of Natural and Biomimetic Drugs, Peking University, Beijing, China; ^2^Department of Molecular and Cellular Pharmacology, School of Pharmaceutical Sciences, Peking University, Beijing, China

**Keywords:** ischemic heart failure, left anterior descending artery, matrix-assisted laser desorption/ionization–mass spectrometry imaging, phospholipid, Shenfu injection

## Abstract

**Objectives:** We aimed to evaluate the effect of Shenfu injection in a rat model of ischemic heart failure and explore its mechanism.

**Methods:** A rat model of ischemic heart failure after myocardial infarction was established by ligating the left anterior descending coronary artery. Forty-eight hours after surgery, the rats were intraperitoneally administered Shenfu injection for 7 weeks. Then, left ventricular fractional shortening and left ventricular ejection fraction were measured using transthoracic echocardiography, whereas heart rate and left ventricular end-diastolic pressure were measured using a MD3000 biosignal acquisition and processing system. The hearts and lungs of the rats were excised and weighed to measure the heart and lung weight indexes. In addition, cardiac histopathological changes were observed *via* hematoxylin–eosin and Masson’s trichrome staining, and serum cardiac troponin content was detected using a cardiac troponin ELISA kit. Furthermore, matrix-assisted laser desorption/ionization–mass spectrometry imaging was used to detect the levels and distribution of small molecules in the hearts of rats with ischemic heart failure.

**Results:** We found that Shenfu injection can significantly increase left ventricular fractional shortening and left ventricular ejection fraction in rats with ischemic heart failure and significantly reduce the left ventricular end-diastolic pressure, heart and lung weight indexes, and cardiac troponin content; improve cardiac tissue morphology; and reduce infarct size. In addition, the matrix-assisted laser desorption/ionization–mass spectrometry imaging results demonstrated that 22:6 phospholipids were predominately distributed in the non-infarct zone, whereas 20:4 phospholipids tended to concentrate in the infarct zone. Shenfu injection significantly reduced taurine, glutathione, and phospholipids levels in the hearts of rats with ischemic heart failure and primarily changed the distribution of these molecules in the non-infarct zone.

**Conclusion:** Shenfu injection induced obvious myocardial protective effects in rats with ischemic heart failure by stimulating antioxidation and changing the phospholipid levels and distribution.

## Introduction

Myocardial ischemia develops when coronary arteries, the major route of blood supply to the heart, are damaged. Recently, ischemic heart disease has become one of the most serious cardiovascular diseases worldwide, and ischemic heart failure (IHF) is an important cause of death (Liu et al., 2018). When ischemia develops in the heart, blood supply becomes limited, thereby accelerating the insufficiency of energy. Consequently, myocardial cells become dysfunctional and damaged (Liu et al., 2018), eventually leading to myocardial infarction (MI). In addition, myocardial remodeling after long-term MI leads to the deposition of fibrous tissue in the myocardium and impaired function, further leading to heart failure (Takemura et al., 2018). Left ventricular remodeling caused by MI accounts for approximately 70% of the 5 million cases of heart failure in the United States (Benjamin et al., 2018), and the mortality rate of heart failure after MI remains high in China and increases with increasing disease duration (Chen et al., 2017).

From short-term hemodynamic/pharmacological measures to long-term restorative strategies aimed at altering the biological properties of failing hearts, the treatment of IHF has undergone a noteworthy transformation since the 1990s (Yancy et al., 2013). Drugs targeting the renin–angiotensin–aldosterone system and the parasympathetic nervous system are attracting increasing attention, and positive inotropic drugs, beta-blockers [e.g., carvedilol (CAR)], angiotensin-converting enzyme inhibitors, and diuretics have become common treatments for IHF (Talameh and Lanfear, 2012; Yancy et al., 2013). Previous studies have confirmed that beta-blockers (such as CAR, etc.) can effectively treat heart disease, including IHF (Hjalmarson and Waagstein, 1994).

Shenfu injection is an injectable Chinese herbal medicine derived from Shenfu decoction. It is exclusively produced by Huarun Sanjiu Pharmaceutical Co., Ltd. (Sichuan, China). Shenfu injection is a refined suspension and mainly contains ginsenosides and aconitines; its primary effects include diuresis, reduction of myocardial oxygen consumption, alleviation of ischemia-reperfusion injury, and inhibition of inflammatory reactions (Wang et al., 2009; Guo and Li, 2013). Therefore, Shenfu injection can be used to treat IHF.

Matrix-assisted laser desorption/ionization–mass spectrometry imaging (MALDI–MSI) facilitates the *in situ* analysis of compounds without the need for labeling in two-dimensional biological tissue sections, thereby revealing the molecular information of compounds; the spatial coordinates and distribution of compounds in the tissue under study are linked. Using 1,5-diaminonaphthalene hydrochloride as a substrate, MALDI–MSI can detect various small molecules, including metal ions, tricarboxylic acid (TCA) cycle- and energy metabolism-related molecules, small endogenous antioxidants, and phospholipids, in heart tissue (Liu et al., 2014; Liu et al., 2017). These molecules have important implications for studying the effects of Shenfu injection on oxidative stress and myocardial remodeling in rats with IHF.

The present study confirmed the effects of Shenfu injection on myocardial injury in rats with IHF and elucidated its mechanism of action.

## Materials and Methods

### Drug and Reagents

Shenfu injection (production batch no.: 17040401001) was provided by Huarun Sanjiu Pharmaceutical Co., Ltd. (Sichuan, China). Shenfu injection is extracted from two Chinese medicinal materials: red ginseng root (*Panax ginseng* C. A. Mey) and aconite tuber (*Aconitum carmichaeli* Debeaux). A flow chart of the Shenfu injection preparation process has been presented in **Supplementary Figure 1**. Briefly, red ginseng root and aconite tuber were separately soaked and concentrated into solutions and mixed in a ratio of 1:2 by volume to obtain Shenfu mixture. This mixture was then refined into Shenfu injection, followed by filling, sterilization, and quality evaluation. Shenfu injection was analyzed using high-performance liquid chromatography (HPLC) (**Supplementary Figure 2**) and was determined to mainly contain ginsenosides and aconitines [Ginsenoside Rg1 (0.13 mg/ml), ginsenoside Re (0.12 mg/ml), ginsenoside Rf (0.07 mg/ml), ginsenoside Rb1 (1.4 mg/ml), ginsenoside Rc (0.08 mg/ml), ginsenoside Rg2 (0.04 mg/ml), benzoylmesaconine (1.60 µg/ml), benzoylaconine (0.39 µg/ml), and benzoylhypacoitine (0.95 µg/ml)]. Of all these components, ginsenoside Rg1, ginsenoside Re, ginsenoside Rb1, and benzoylmesaconine were required to meet the quality standards of Shenfu injection (**Supplementary Table 1**), which was in line with the standards of the China Food and Drug Administration (Standard test nos.: WS3-B-3427-98-2013 and P. ZL. 205-001).

Ginsenosides were chromatographically analyzed using a Waters Symmetry Shield™ RP18 column (4.6 × 250 mm; 5.0 µm); the column temperature was maintained at 40°C. The mobile phases (A: Acetonitrile; B: H_2_O) were 0%–10% A for 0–30 min, 10%–23% A for 30–40 min, 23% A for 40–50 min, and 23%–60% A for 50–80 min. Flow rate was maintained at 1.0 ml/min, and the detection wavelength for HPLC analysis was set at 203 nm. The analysis of aconitines was performed using a Thermo Hypersil BDS C18 column (4.6 × 250 mm; 5.0 µm) and column temperature was maintained at 30°C. The mobile phases [A: 0.2% Triethylamine (adjust pH to 5.3 with glacial acetic acid); B:CH_3_CN] were 70% A for 0–30 min and 45% A for 30–40 min. Flow rate was maintained at 1.0 ml/min, and the detection wavelength for the HPLC analysis was set at 235 nm.

CAR tablets were purchased from Qilu Pharmaceutical Co., Ltd. (Shandong, China). Heparin sodium was purchased from Beijing Suolaibao Technology Co., Ltd. (Beijing, China). Cardiac troponin T (cTnT) test kit was purchased from Wuhan Huamei Bioengineering Co., Ltd. (Wuhan, China). 1,5-Diaminonaphthalene was purchased from Sigma–Aldrich (St. Louis, Missouri, USA). Hematoxylin, eosin, Ponceau, and aniline blue were provided by Servicebio (Wuhan, China).

### Animals

One hundred male age-matched Sprague–Dawley rats weighing 230–260 g were purchased from Beijing Vital River Laboratory Animal Technology Co., Ltd. [Beijing, China; license no.: SCXK (Beijing) 2016-0011]. The rats were labeled, weighed, caged, and permitted to eat and drink *ad libitum*. The temperature and humidity in the cage were maintained at 22°C–24°C and 50%–60%, respectively. The rats were maintained under a 12-h light/dark cycle and were allowed an acclimatization period of 1 week before surgery. All animal experiments were approved by the Peking University Biomedical Ethics Committee (Beijing, China; approval no.: LA2017282). The animal experimenters hold employment certificates from the Department of Laboratory Animal Science, Peking University Health Science Center, and have passed the laboratory entrance test held by Peking University School of Pharmaceutical Sciences.

### IHF Model

The rats were intraperitoneally anesthetized with 0.5% pentobarbital sodium (1 ml/100 g), placed in the supine position, and provided positive-pressure ventilation using a small animal ventilator (frequency: 80 beats/min; respiratory ratio: 1:1; ventilation: 3 ml/100 g). Thereafter, the rats’ limbs were connected to an electrocardiogram electrode and electrocardiography was performed (**Supplementary Figure 3A**). Next, thoracotomy was performed, exposing the heart, and the left anterior descending coronary artery was threaded and ligated, thereby producing a model of IHF. The procedure was considered successful when ST segment elevation was observed on the electrocardiogram (**Supplementary Figure 3B**). The normal (N) group received no treatment, whereas the sham (S) group underwent surgery without ligation of the left anterior descending coronary artery (Smith et al., 2003; Lv et al., 2016).

### Grouping and Administration

**Figure 1:**
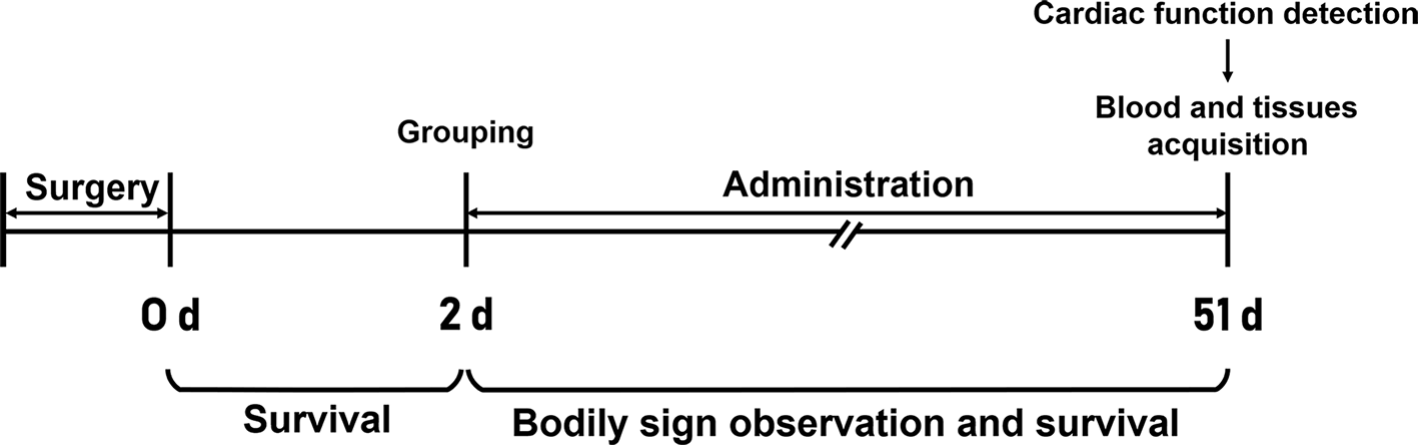
Flow chart of the study workflow.

The surviving rats with ligation of the left anterior descending artery were randomly divided into the model (MOD), CAR [10 mg/kg (Grandinetti et al., 2019), 6 ml/kg], and Shenfu injection groups [L, M, and H groups receiving Shenfu injection at concentrations of 3, 6, and 12 ml/kg, respectively (corresponding to clinical dosages of 36, 72, and 144 ml/70 kg, respectively)] 48 h after surgery. The CAR group (n = 9) received the drug intragastrically, whereas the L, M, and H groups received Shenfu injection intraperitoneally. The N, S, and MOD groups received normal saline intraperitoneally at 12 ml/kg/day. Then, body signs and survival of all rats were observed and recorded. A flow chart of the study workflow is illustrated in **Figure 1**.

### Echocardiography and Hemodynamics

Seven weeks later, the rats were anesthetized with 2% isoflurane and echocardiographic examination was performed using a Vevo2010 ultra-high–resolution small animal imaging ultrasound system (Visual Sonics, Canada). Left ventricular end-diastolic diameter (LVEDD), left ventricular end-systolic diameter (LVESD), left ventricular fractional shortening (LVFS), and left ventricular ejection fraction (LVEF) were also measured. Thereafter, the right common carotid artery was used for catheter intubation, and the arterial catheter was then connected to a MD3000 biosignal acquisition and processing system for hemodynamic testing, including measurements of left ventricular end-diastolic pressure (LVEDP) and heart rate (HR).

### Serum cTnT Measurement

After hemodynamic testing, blood (1 ml) was drawn from the abdominal aorta each rat and placed in a centrifuge tube. The blood samples were then centrifuged at 3,000 rpm for 15 min to obtain serum. The cTnT concentration in serum was determined using the cTnT test kit.

### Histopathological Examination

After obtaining the blood, the hearts of four rats from each group were quickly separated, excised, and fixed in 4% paraformaldehyde for 48 h to prepare a 4-µm-thick paraffin section for hematoxylin–eosin (HE) and Masson’s trichrome staining, which were performed to observe cardiac histopathological changes under a light microscope; myocardial infarct size (IS) was calculated using the following formula: IS (%) = midline infarct length/left ventricular midline circumference × 100% (Takagawa et al., 2007). In addition, the hearts and lungs of the other rats were obtained and cleaned. Heart weight index (HWI) and lung weight index (LWI) of the rats in each group were calculated using the following formula: organ index = organ wet weight (mg)/body weight of the rat on the day of sacrifice (g).

### Matrix-Assisted Laser Desorption/Ionization–Mass Spectrometry Imaging

After obtaining the blood, the hearts of three rats each from the H, S, and MOD groups were quickly separated, excised, snap-frozen in liquid nitrogen and stored at −80°C for MALDI–MSI.

The snap-frozen hearts were fixed to the sample holder using ultrapure water, and 10-µm-thick transverse slices were prepared using a cryostat microtome (Scotsman Jencons, Germany) at −17°C. The distance from the slice position to the apex was 2/5^th^ of the vertical length of the heart. The tissue slices were then transferred onto indium tin oxide slides (Bruker Daltonics, Germany) and dried in a vacuum pump for 30 min. Matrix spraying and MALDI–MSI were then conducted, as previously described (Liu et al., 2014; Liu et al., 2017). Molecular spectrum peaks were selected in the MALDI–MSI results, and statistical analysis was performed using the SCiLS Lab software based on the normalization of total ion chromatography.

### Statistical Analysis

Data were expressed as mean ± standard error of the mean. Survival curve results were analyzed using Student’s *t*-test (two-tailed), and the other results were compared using one-way analysis of variance. All statistical data were analyzed using the GraphPad Prism 6 software. *P* < 0.05 was considered statistically significant.

## Results

### Effect of Shenfu Injection on Body Signs and Survival of the Rats With IHF

After treatment, the body signs and survival of rats were recorded. Compared with the rats in the S group, those in the MOD group consumed less food and water and exhibited less activity; moreover, some animals in the MOD group displayed dyspnea. After treatment, the rats were not easily scared and exhibited less dyspnea and more night activity.

Survival curves were drawn for the rats in each group, except for nine that died during surgery. Compared with that in the S group, the survival rate significantly decreased in the MOD group in the early phase of myocardial ischemia (*P* < 0.05) from post-surgery to pre-therapy (**Supplementary Figure 4A**). After treatment, the survival rate of the MOD group was significantly reduced (<60%; *P* < 0.05); in contrast, the L, M, and H groups had survival rates >80% (**Supplementary Figure 4B**).

### Shenfu Injection Improves Left Ventricular Systolic Function of the Rats With IHF

**Figure 2:**
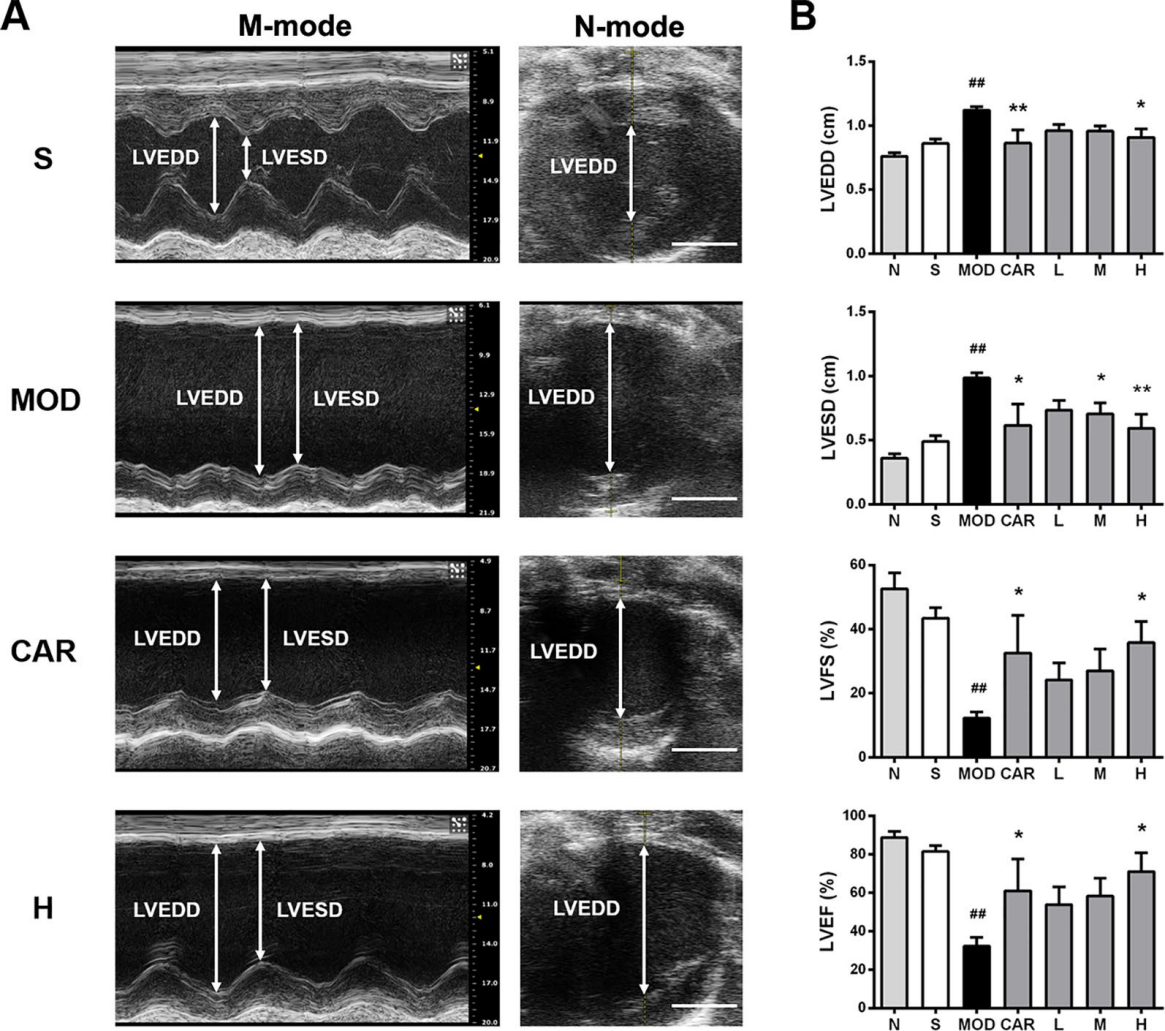
Effect of Shenfu injection on the echocardiographic characteristics of the rats with ischemic heart failure. **(A)** M-mode and B-mode echocardiographic images of the hearts of rats in the S, MOD, CAR, and H groups. **(B)** Left ventricular end-diastolic diameter (LVEDD), left ventricular end-systolic diameter (LVESD), left ventricular fraction shortening, and left ventricular ejection fraction of the rats with IHF. Arrows demarcate the LVESD and LVEDD dimensions; scale bar, 5 mm. N, normal (n = 3); S, sham (n = 3); MOD, model (n = 4); CAR, carvedilol (n = 4); L, 3 ml/kg Shenfu injection (n = 5); M, 6 ml/kg Shenfu injection (n = 4); H, 12 ml/kg Shenfu injection (n = 4). Data are expressed as the mean ± standard error of the mean. ^##^ *P* < 0.05 vs. S group; *P < 0.05, **P < 0.01 vs. MOD group.

Left ventricular systolic function, as assessed using LVFS and LVEF, was impaired in the rats with IHF, with the changes matched to elevations of LVESD and LVEDD (Smith et al., 2005; Xing et al., 2014). As shown in **Figure 2**, LVEDD and LVESD were higher in the MOD group than in the S group (both *P* < 0.01); in contrast, LVFS and LVEF were lower in the MOD group than in the S group (*P* < 0.01 and *P* < 0.001, respectively). After treatment with Shenfu injection, LVEDD and LVESD were significantly decreased in the H group (*P* < 0.05 and *P* < 0.01, respectively), whereas LVFS and LVEF were obviously increased (both *P* < 0.05). In addition, CAR obviously lowered LVEDD and LVESD (*P* < 0.01 and *P* < 0.05, respectively) and improved LVFS and LVEF (both *P* < 0.05).

### Shenfu Injection Enhances Left Ventricular Diastolic Function of the Rats With IHF

**Figure 3:**
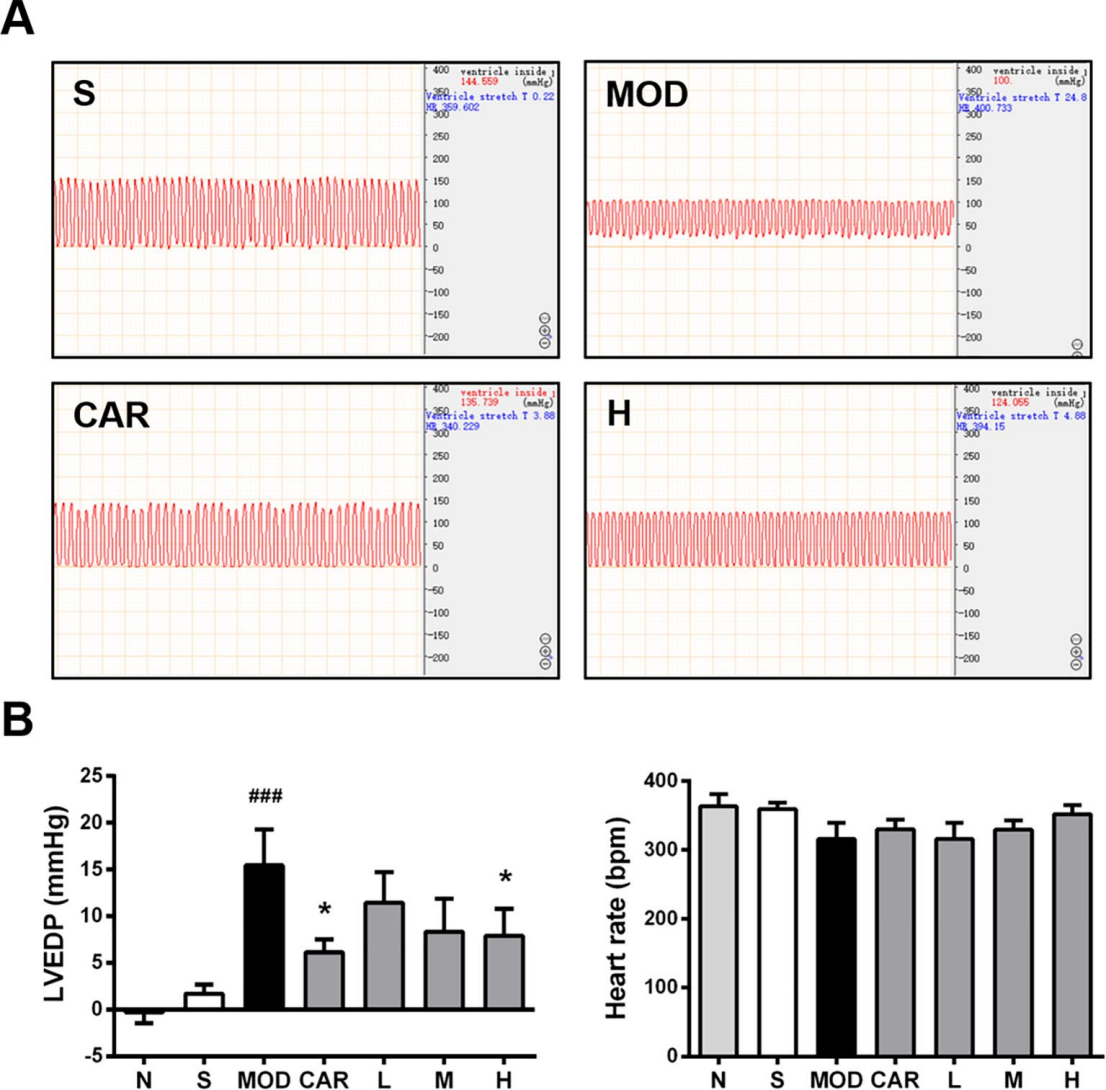
Effect of Shenfu injection on the hemodynamics of the rats with ischemic heart failure. **(A)** Hemodynamic diagrams in the hearts of the rats in the S, MOD, CAR, and H groups. **(B)** Left ventricular end-diastolic pressure (LVEDP) and heart rate in the rats with IHF. N, normal (n = 7); S, sham (n = 11); MOD, model (n = 7); CAR, carvedilol (n = 7); L, 3 ml/kg Shenfu injection (n = 7); M, 6 ml/kg Shenfu injection (n = 7); H, 12 ml/kg Shenfu injection (n = 7). Data are expressed as the mean ± standard error of the mean. ^###^ *P* < 0.001 vs. S group; *P < 0.05 vs. MOD group.

Hemodynamic results indicating the changes of LVEDP and HR are shown in **Figure 3A**. L VEDP was inversely correlated with left ventricular diastolic function; moreover, LVEDP was significantly increased in the MOD group (*P* < 0.001) (**Figure 3B**), and 12 mg/kg Shenfu injection obviously reduced LVEDP (*P* < 0.05) (**Figure 3B**). In addition, CAR reduced LVEDP in the rats with IHF (*P* < 0.05) (**Figure 3B**). Thereafter, there was no significant difference in the HR among the S, MOD, and Shenfu injection groups (**Figure 3B**), consistent with prior findings (Smith et al., 2005).

### Shenfu Injection Attenuates Abnormal Hypertrophy of the Heart and Lung of the Rats With IHF

**Figure 4:**
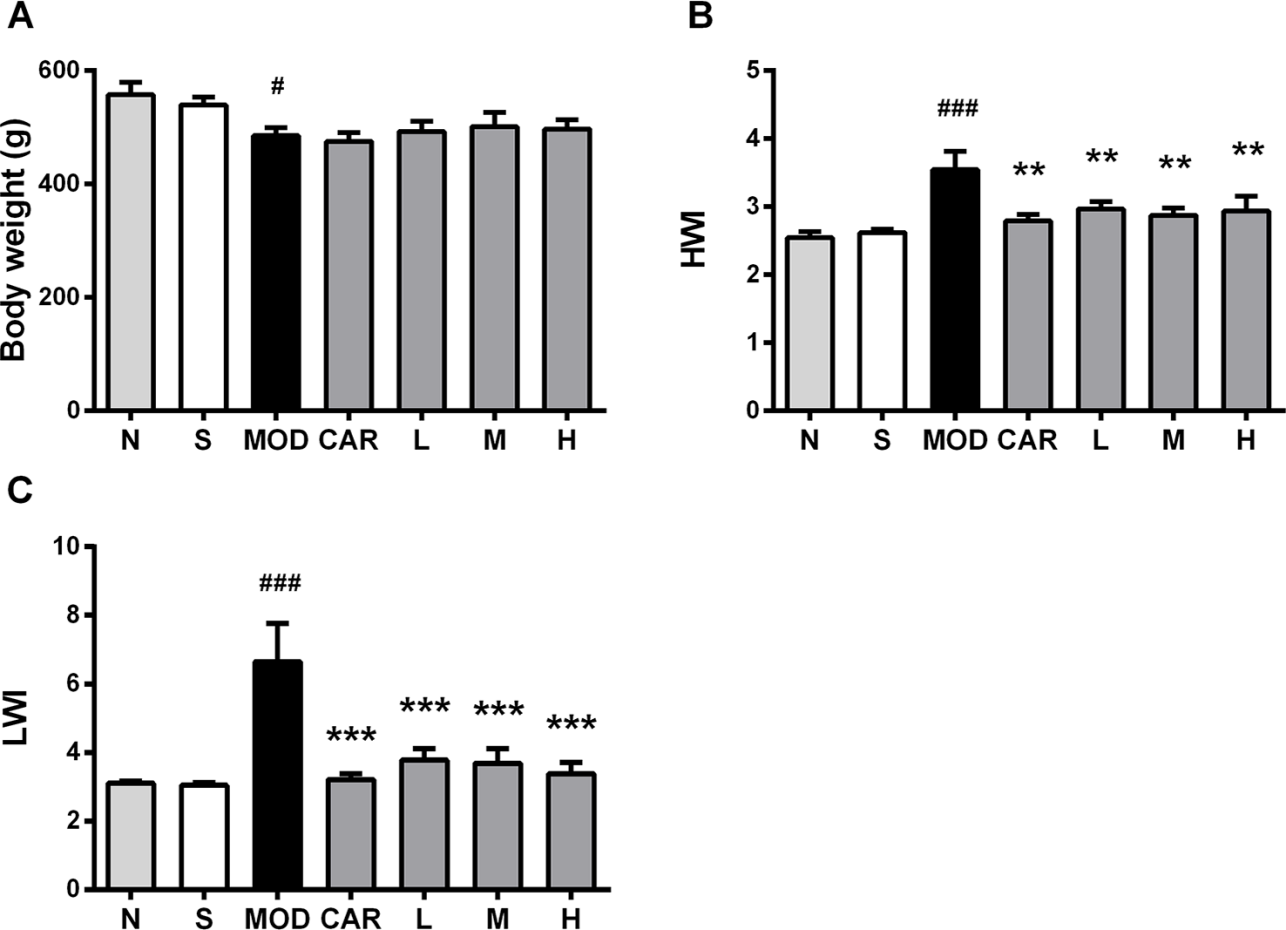
Effect of Shenfu injection on body weight of the rats with ischemic heart failure. **(A**–**C)** Body weight, heart weight index, and lung weight index. N, normal (n = 7); S, sham (n = 11); MOD, model (n = 7); CAR, carvedilol (n = 7; L, 3 ml/kg Shenfu injection (n = 8); M, 6 ml/kg Shenfu injection (n = 7); H, 12 ml/kg Shenfu injection (n = 7). Data are expressed as mean ± standard error of the mean. ^#^ *P* < 0.05, ^###^ *P* < 0.001 vs. S group; **P < 0.01, ***P < 0.001 vs. MOD group.

HWI and LWI, which are associated with pathological conditions, such as cardiac hypertrophy and pulmonary blood stasis, are significantly greater in rats with IHF during the development of IHF (Smith et al., 2003). Compared with the S group, the MOD group exhibited decreased body weight (*P* < 0.05) (**Figure 4A**) and increased HWI and LWI (both *P* < 0.001) (**Figures 4B, C**). HWI (all *P* < 0.01) (**Figure 4B**) and LWI (all *P* < 0.001) (**Figure 4C**) were significantly improved in all Shenfu injection groups after treatment. In addition, treatment with CAR significantly improved HWI and LWI in the rats with IHF (*P* < 0.01 and *P* < 0.001, respectively) (**Figures 4B, C**).

### Effect of Shenfu Injection on Heart Histopathology of the Rats With IHF

#### Shenfu Injection Inhibits Myocardial Damage

**Figure 5:**
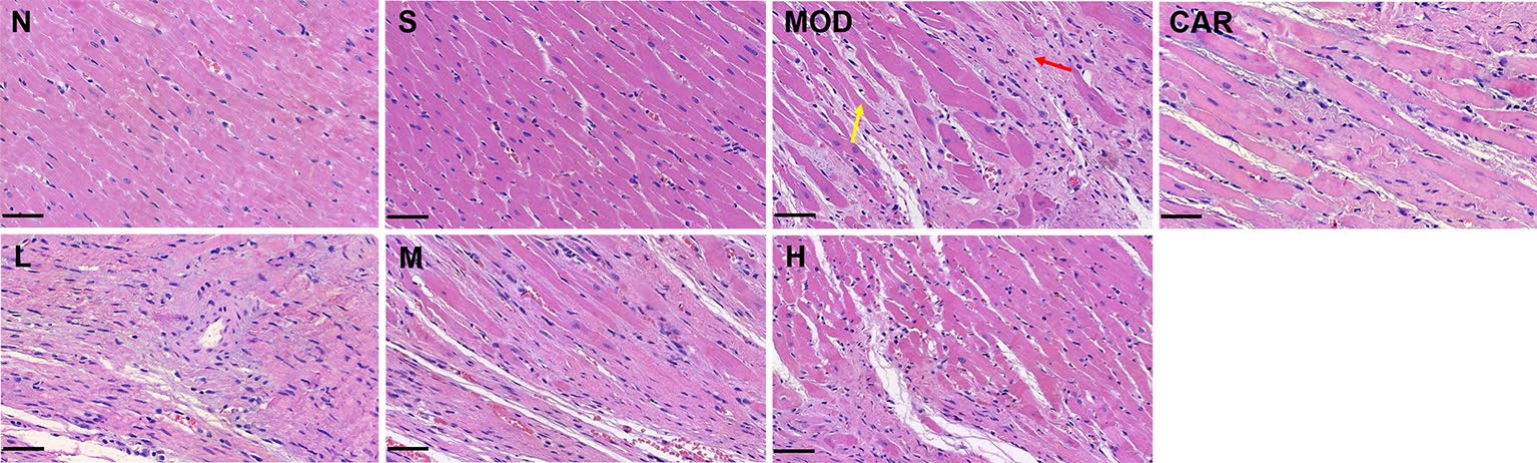
Photomicrographs of heart sections stained with hematoxylin–eosin in each group after coronary artery ligation (close to the coronary vein, 200×). Scale bar, 50 μm in each panel. The yellow arrow denotes damaged myocardial fibers, whereas the red one indicates inflammatory cell infiltration. N, normal; S, sham; MOD, model; CAR, carvedilol; L, 3 ml/kg Shenfu injection; M, 6 ml/kg Shenfu injection; H, 12 ml/kg Shenfu injection.

As demonstrated in **Figure 5**, myocardial tissue lesions of rats in the MOD group were primarily located in the middle of the outer left ventricle wall wherein damaged myocardial fibers (yellow arrow), exaggerated nuclei with a deep color, and severe inflammatory cell infiltration (red arrow) were observed. After the administration of 12 mL/kg Shenfu injection, the extent of myocardial fiber damage and inflammatory cell infiltration was improved.

#### Shenfu Injection Decreases Myocardial IS

**Figure 6:**
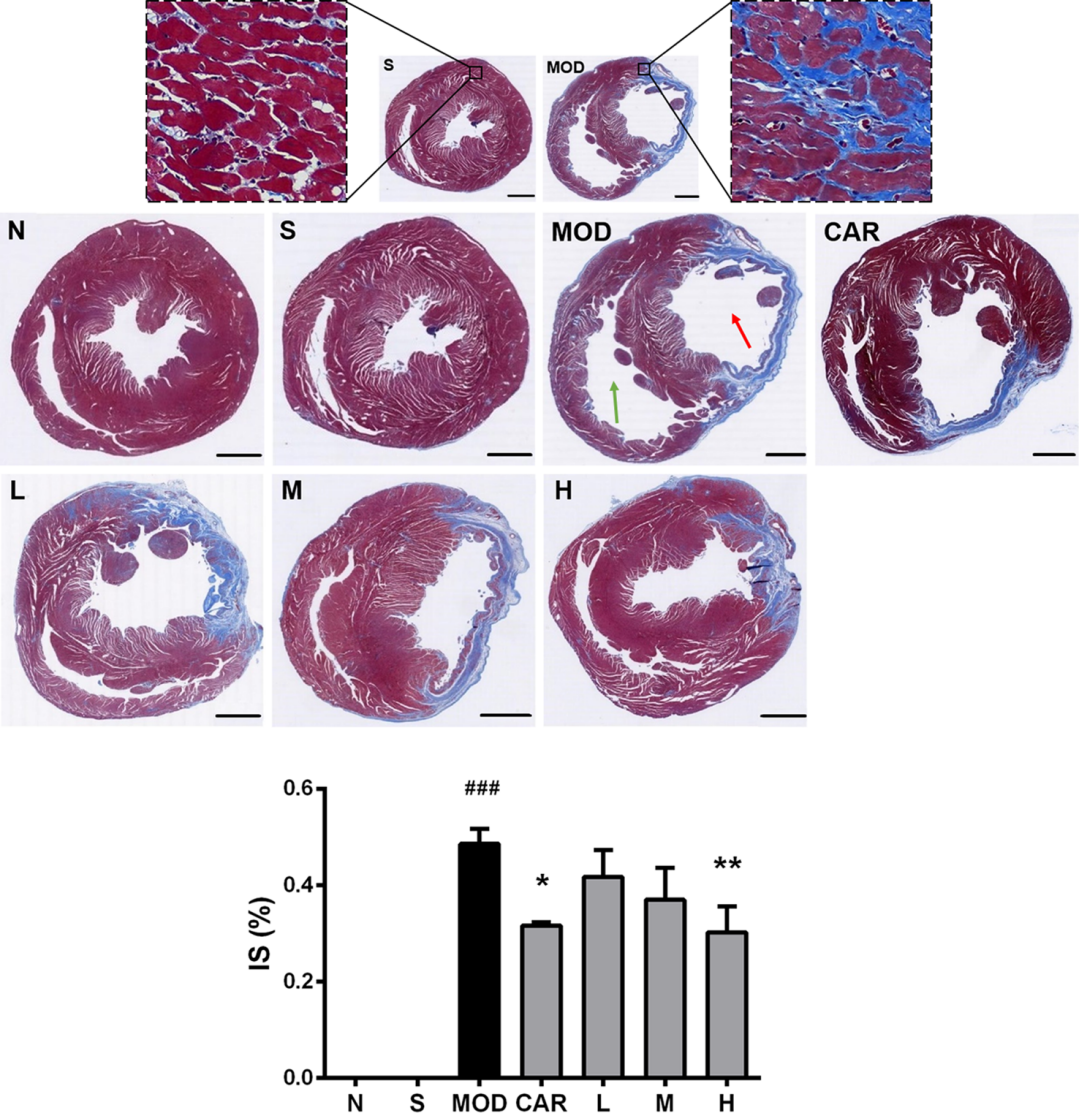
Photomicrographs of Masson’s trichrome-stained heart sections of rats from each group after coronary artery ligation. The magnification of the cross-sectional photomicrographs is 5.1× (scale bar, 2000 μm in each panel). The magnification of the local enlarged photomicrographs near the coronary vein is 200× (scale bar, 50 μm in each panel). The red arrow indicates the left ventricle, whereas the green one indicates the right ventricle. The infarct size was analyzed using Image-Pro Plus. N, normal (n = 4); S, sham (n = 4); MOD, model (n = 5); CAR, carvedilol (n = 4); L, 3 ml/kg Shenfu injection (n = 4); M, 6 ml/kg Shenfu injection (n = 4); H, 12 ml/kg Shenfu injection (n = 4). Data are expressed as mean ± standard error of the mean. ^###^ *P* < 0.001 vs. S group; *P < 0.05, **P < 0.01 vs. MOD group.

As shown in **Figure 6**, large amounts of blue collagen fibers appeared in the myocardial tissue lesions in the left ventricles (red arrow) of rats in the MOD group; compensatory hypertrophy and enlarged ventricular chamber were observed in the right ventricle (green arrow). After the administration of Shenfu injection or CAR, the amount of blue collagen fibers was reduced, and the left and right ventricular walls were more stable. Furthermore, 12 mg/kg Shenfu injection or CAR significantly decreased the size of the myocardial tissue lesions (*P* < 0.01 and *P* < 0.05, respectively).

### Shenfu Injection Reduces Serum cTnT Levels of the Rats With IHF

In rats, myocardial ischemic injury leads to an increase in the serum cTnT levels (Wei et al., 2015). As shown in **Supplementary Figure 5**, compared with that in the S group, the increase in serum cTnT levels in the MOD group (*P* < 0.01) was significantly inhibited by 12 ml/kg Shenfu injection and CAR (both *P* < 0.05); however, 6 ml/kg Shenfu injection did not affect the serum cTnT levels.

### Effect of Shenfu Injection on the Content and Distribution of 80–1000-Da Molecules in the Hearts of the Rats With IHF

Using MALDI–MSI, significant changes in the levels and distribution of some small molecules, including endogenous antioxidant molecules (taurine, glutathione, and ascorbic acid), phospholipids [phosphatidic acid (PA), phosphatidylethanolamine (PE), and phosphatidylinositol (PI)], and TCA cycle- and energy metabolism-related molecules (aspartate, N-acetyl--aspartate, and adenosine), were observed in the whole transverse heart tissue slices of the rats with IHF.

**Figure 7:**
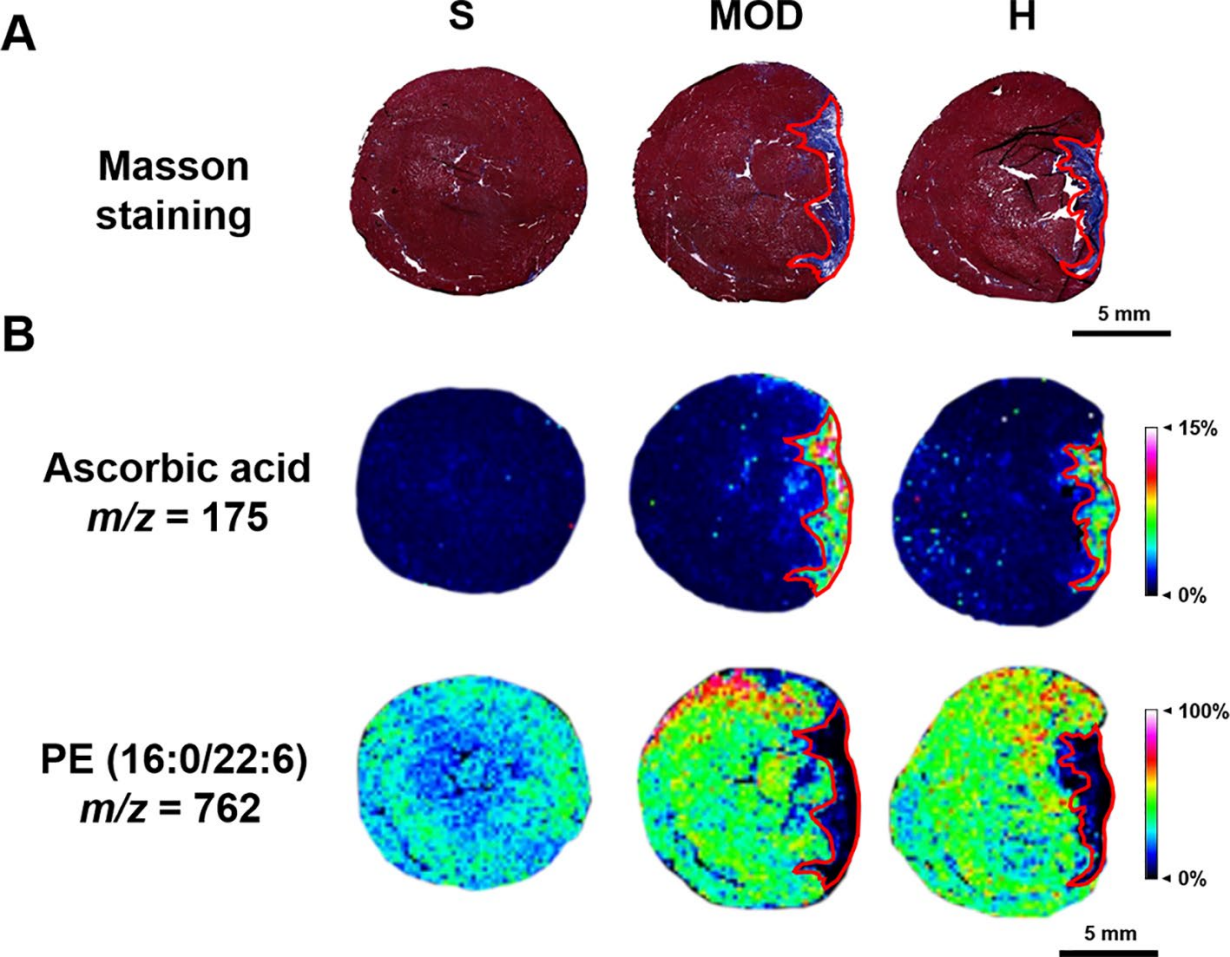
Infarct zone of the matrix-assisted laser desorption/ionization–mass spectrometry imaging (MALDI–MSI) results (marked with red coils). **(A)** Masson staining of the adjacent heart slice. **(B)** The MALDI-MSI results of ascorbic acid and PE (16:0/22:6). The spatial resolution is 200 μm (scale bar, 5 mm). S, sham; MOD, model; H, 12 ml/kg Shenfu injection.

Based on the MALDI–MSI results of ascorbic acid and PE (16:0/22:6) in the heart slices and Masson’s trichrome-stained images of the adjacent slices, the infarct zone was located using the MSI images and marked with red coils, as shown in **Figure 7**.

#### Shenfu Injection Changes Antioxidant Molecules Levels in the Rats With IHF

**Figure 8:**
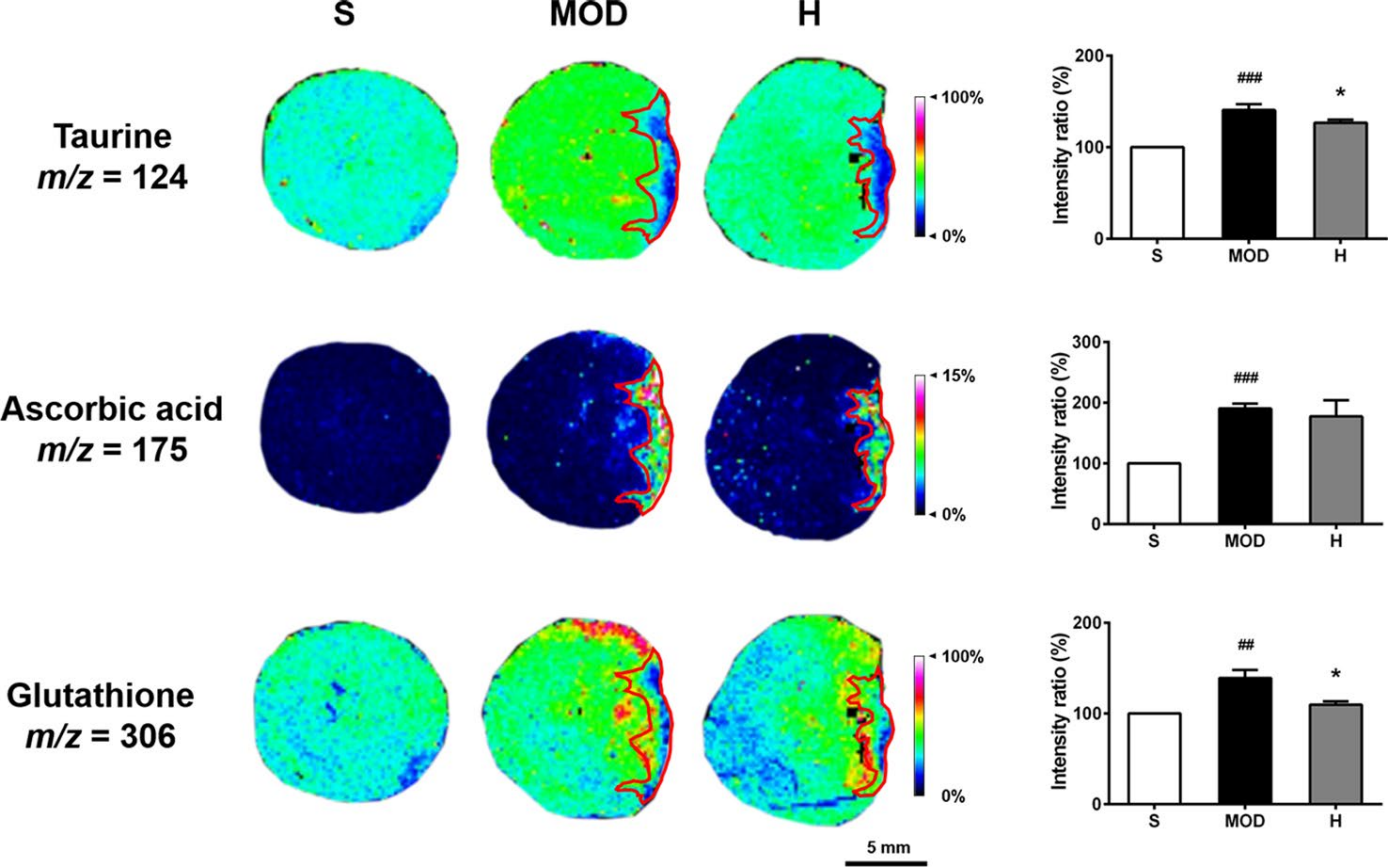
Effect of Shenfu injection on antioxidant molecules in the hearts of rats with ischemic heart failure. Intensity ratios of molecules were analyzed using the SCiLS Lab software in a normalized manner. Spatial resolution is 200 μm (scale bar, 5 mm). S, sham; MOD, model; H, 12 ml/kg Shenfu injection. Data are presented as mean ± standard error of the mean; n = 3 per group. ^##^ *P* < 0.01, ^###^ *P* < 0.001 vs. S group; *P < 0.05 vs. MOD group.

Taurine, ascorbic acid, and glutathione levels were significantly increased in the whole hearts of the rats in the MOD group than in the hearts of those in the S group (*P* < 0.001, *P* < 0.001, and *P* < 0.01, respectively). Shenfu injection reduced the glutathione and taurine levels (both *P* < 0.05) compared with those in the MOD group; however, it had no effect on ascorbic acid, as shown in **Figure 8**. In addition, in the MOD group, ascorbic acid was primarily distributed in the infarct zone, whereas taurine and glutamine were evenly distributed in the infarct as well as non-infarct zones; Shenfu injection primarily changed the distribution of taurine and glutamine in the non-infarct zone.

#### Shenfu Injection Regulates Phospholipids in the Rats With IHF

**Figure 9:**
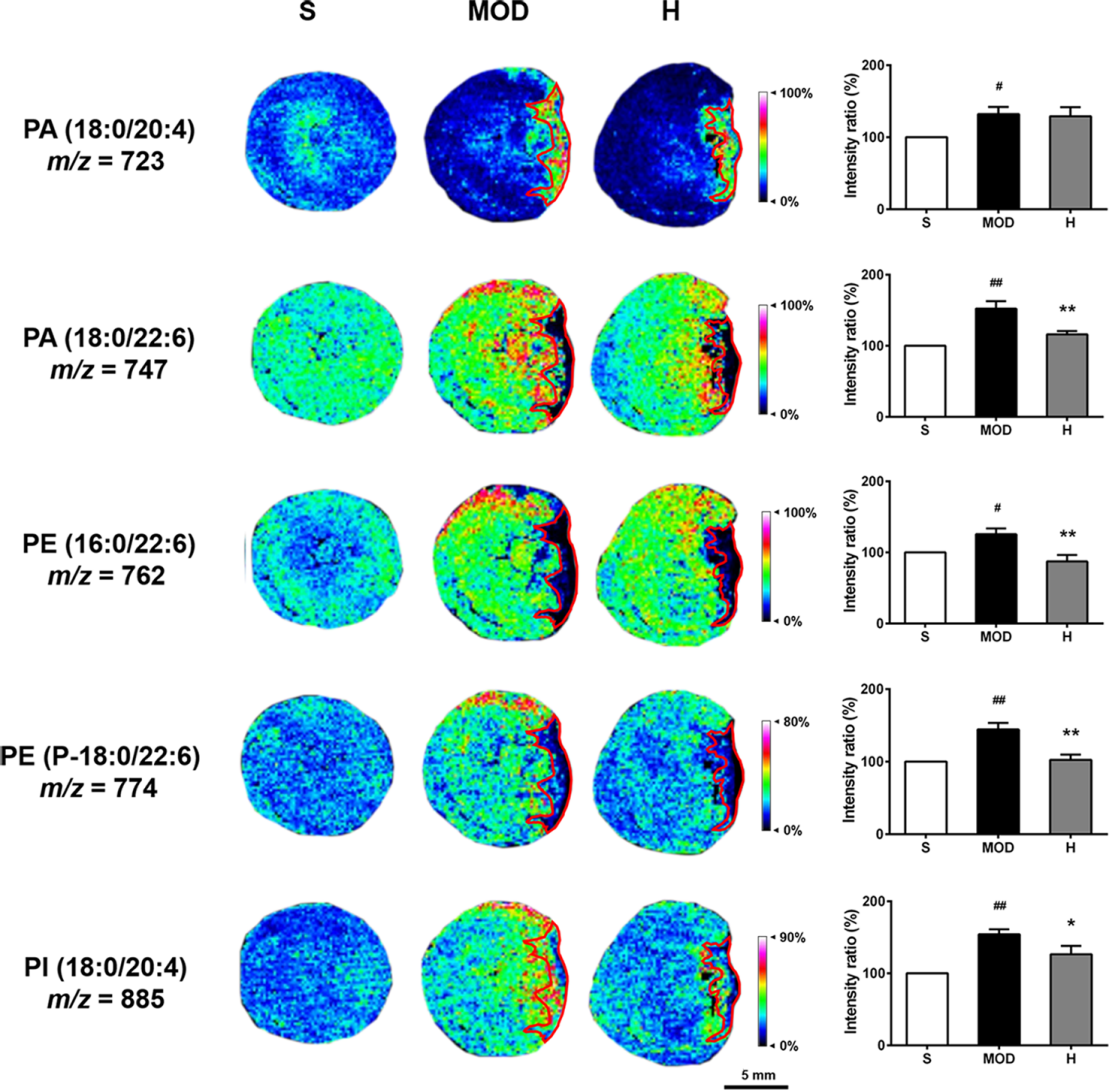
Effect of Shenfu injection on phospholipids in the hearts of rats with ischemic heart failure. Intensity ratios of molecules were analyzed using SCiLS Lab software in a normalized manner. The spatial resolution is 200 μm (scale bar, 5 mm). S, sham; MOD, model; H, 12 ml/kg Shenfu injection. Data are presented as mean ± standard error of the mean; n = 3 per group. ^#^ *P* < 0.05, ^##^ *P* < 0.01 vs. S group; *P < 0.05, **P < 0.01 vs. MOD group.

As shown in **Figure 9**, the PA (18:0/20:4), PA (18:0/22:6), PE (16:0/22:6), PE (P-18:0/22:6), and PI (18:0/20:4) levels were increased in the hearts of the rats in the MOD group than in the hearts of those in the S group (*P* < 0.05, *P* < 0.01, *P* < 0.05, *P* < 0.01, and *P* < 0.01, respectively); in contrast, Shenfu injection reduced the PA (18:0/22:6), PE (16:0/22:6), PE (P-18:0/22:6), and PI (18:0/20:4) levels (*P* < 0.01, *P* < 0.01, *P* < 0.01, and *P* < 0.05, respectively), with no significant impact on PA (18:0/20:4). Furthermore, PA (18:0/20:4), PE (16:0/22:6), and PE (P-18:0/22:6) in the hearts of the rats in the MOD group were observed to be primarily located in the non-infarct zone, and PA (18:0/20:4) was located in the infarct zone. Shenfu injection predominately changed the distribution of phospholipids in the non-infarct zone.

#### Shenfu Injection has no Effect on TCA Cycle- and Energy Metabolism-Related Molecules in the Rats With IHF

As shown in **Supplementary Figure 6**, the aspartate and N-acetyl-aspartate levels were increased in the MOD group than in the S group (both *P* < 0.05); in contrast, adenosine levels were decreased in the MOD group than in the S group (*P* < 0.01). Shenfu injection did not reverse these abnormal changes. Further analysis revealed that in the MOD group, aspartic acid and N-acetyl-aspartate were primarily distributed around the infarct zone, and the distribution of adenosine as a downstream product of energy metabolism was changed in the infarct and non-infarct zones.

## Discussion

In the present study, Shenfu injection effectively improved myocardial ischemic injury, enhanced myocardial function, and reduced HWI and LWI levels in rats with IHF that underwent left anterior descending coronary artery ligation. In addition, using MALDI–MSI, it was first discovered that Shenfu injection can reverse the abnormal levels and distribution changes of taurine, glutathione, PA, PE, and PI in the heart caused by IHF, thereby providing new opinions for studies on the links between PA-mediated signaling pathways and cardiac dysfunction in IHF.

The IHF rat model can well simulate clinical IHF caused by coronary artery stenosis (Haidara et al., 2015). The pathogenesis of IHF is extremely complex, involving a series of cell molecular biology, cardiac function, and morphological changes (Takemura et al., 2018). In our study, 12 ml/kg Shenfu injection exerted a cardiotonic effect by dramatically improving myocardial injury to enhance cardiac function and alleviating the occurrence of myocardial hypertrophy and pulmonary congestion; in contrast, 6 ml/kg Shenfu injection had less obvious effects. We subsequently found that Shenfu injection for the treatment of myocardial ischemia in rats was administered at concentrations >6 ml/kg or for periods >7 weeks (Wu et al., 2011; Zheng et al., 2015).

Indeed, previous studies have demonstrated that Shenfu injection could ameliorate cardiac injury by stabilizing cardiac hemodynamic parameters and improving left ventricular function in IHF (Zheng et al., 2015). Furthermore, using MALDI–MS, Zheng et al. (2015) demonstrated that haptoglobin and pentraxin 3 may be inflammatory markers of IHF. In contrast, our study involved a more comprehensive pharmacological evaluation of Shenfu injection and investigated small molecules that potentially play a role in suppressing oxidative stress to protect the myocardium using MALDI–MSI.

Both taurine and glutathione are endogenous antioxidant molecules present in the body and they induce significant effects on myocardial damage. Taurine can inhibit the abnormal increase in reactive oxygen species (ROS) caused by ischemic injury, enhance the activity of antioxidant enzymes, and reduce the levels of inflammatory markers (Shimada et al., 2015; Dallak, 2017). Glutathione directly scavenges ROS and antagonizes oxidative stress associated with excessive glutathionylation of cardiac myosin-binding protein C, which causes myocardial systolic and diastolic dysfunction (Stathopoulou et al., 2016). Based on our study findings, we hypothesize that the overall accumulation of taurine and glutathione in the MOD group is associated with antagonism of oxidative stress and that Shenfu injection can both reduce oxidative stress caused by myocardial ischemia and indirectly reduce glutathionylation to confer myocardial protection; this was predominately observed in the non-infarcted zone.

In addition, PA and PI play important roles as precursors of various glycerophospholipids and are also involved in the transmission of intracellular signals and cellular functions. PE is a neutral phospholipid present in the heart (Nishizuka, 1992; Ammar et al., 2014). Previous studies have revealed that cardiac non-infarct zones exhibit cardiac hypertrophy and imbalance of myocardial interstitial fibrillar collagen synthesis and that infarct zones exhibit apoptosis, necrosis, and scar formation (Liang et al., 2016; Takemura et al., 2018; Wang et al., 2018). The observed distribution of phospholipids in the non-infarct zone of our IHF rat model might be closely associated with myocardial remodeling in this area. Moreover, small amounts of PI (18:0/20:4) and the majority of PA (18:0/20:4) were distributed in the infarct and border zones (Pilla et al., 2015), which might be related to myocardial remodeling in the infarct zone and transition from the non-infarct zone to the infarct zone. In particular, phospholipids with a 22:6 fatty acyl chain were predominately distributed in the non-infarct zone, whereas phospholipids with a 20:4 fatty acyl chain tended to concentrate in the infarct zone. In addition, these phospholipids have many unsaturated fatty acids, which are susceptible to conversion to oxidized phospholipids (OxPLs) by ROS. OxPLs have proinflammatory effects and are associated with many inflammatory diseases, such as metabolic disorders, degenerative diseases, etc. (Freigang, 2016). Therefore, we consider that Shenfu injection can reverse the increase of the levels of various phospholipids caused by myocardial ischemia, which may slow down the myocardial remodeling process in the non-infarct, border, and infarct zones; indirectly affect the overproduction of OxPLs; and inhibit the occurrence of oxidative stress and inflammatory responses. Furthermore, a previous study indicated that defects in PA-mediated signaling pathways might represent a novel mechanism of cardiac dysfunction in IHF (Tappia et al., 2001). Although our study can provide useful insights through the distribution of PA, further investigation is needed.

MALDI–MSI has an obvious advantage in detecting the levels and distribution of cardiac phospholipids. *In vitro* detection of phospholipids using the liquid chromatography–mass spectrometry or electrospray ionization tandem mass spectrometry technique can accurately quantify the level of a certain lipid but not reflect its distribution (Manickaraj et al., 2017; Tang et al., 2018). However, MALDI–MSI can facilitate the high-throughput detection of phospholipids and their distribution *in situ*. Because phospholipids are closely related to the structure and function of the heart, information regarding their distribution is extremely important.

It cannot be denied that this study had some limitations. Measurements were performed at only a single time point (i.e., at the seventh week), for which the results of phospholipids applied for only the seventh week. In addition, because the biochemical and biological functions of phospholipids are unclear, the effects of phospholipids in the course of IHF are uncertain. Therefore, our novel discoveries regarding phospholipids in IHF need to be confirmed through future studies. Furthermore, the adverse reactions of traditional Chinese medicine injections received recognition increasingly, and the causes of adverse reactions related to several factors such as clinical irrational drug use, the patient’s allergic constitution, intravenous drip rate, and use of dosing. The most common adverse reaction of traditional Chinese medicine injections was the “allergic reaction”, which strengthened the research on the clinical skin test of traditional Chinese medicine injections (Liang et al., 2015). If traditional Chinese medicine injections can be used to screen allergic people through skin tests like cephalosporins and penicillin, the safety will be greatly improved. And the State Food and Drug Administration has strengthened the supervision of Chinese medicine injections. In the new medical insurance catalogue 2017, 26 Chinese medicine injections were restricted.

**Figure 10:**
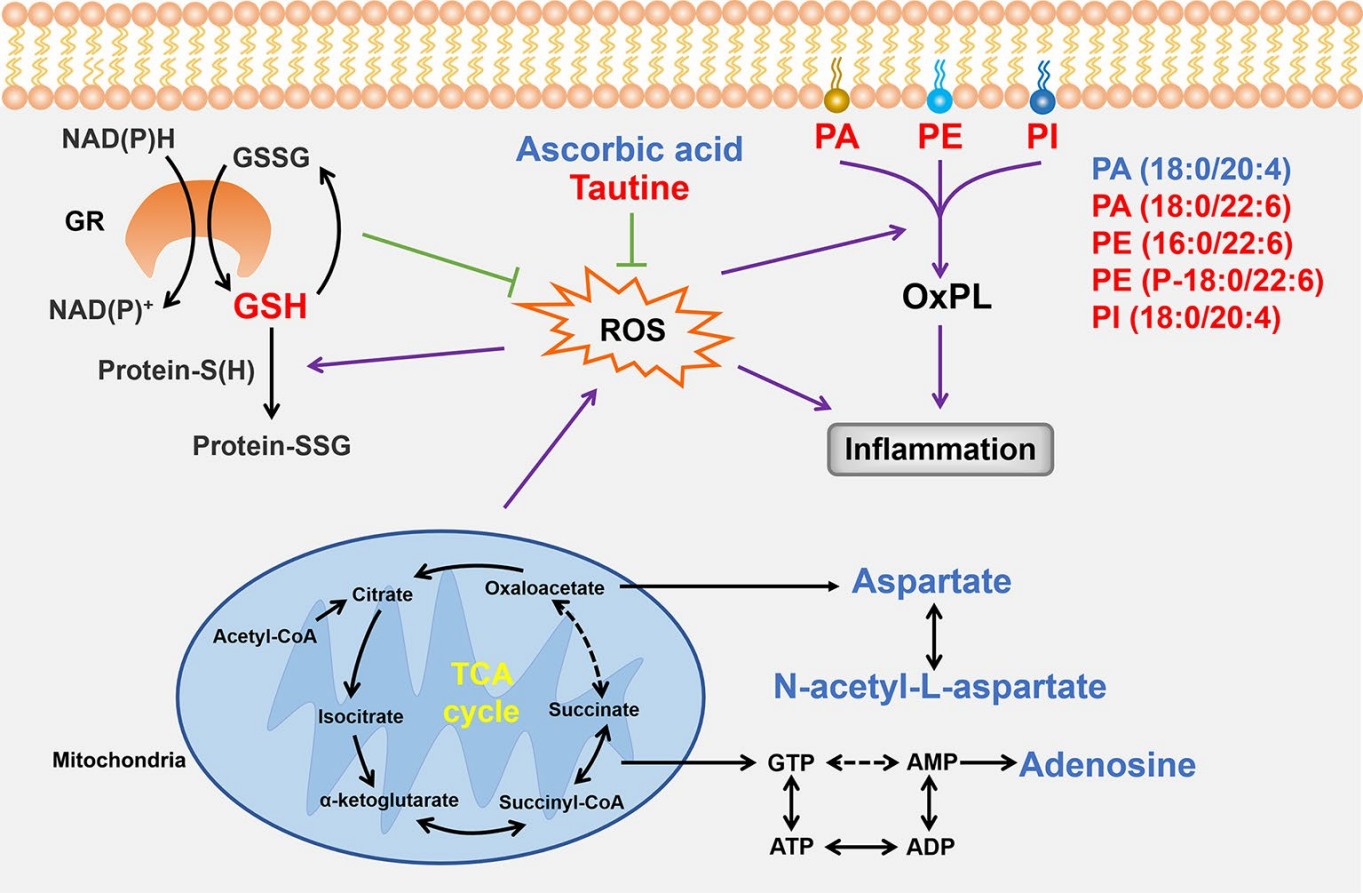
Summary of the relationships between the small molecules found in the present study. Small molecules that Shenfu injection significantly affected are indicated by red font, whereas those that were significantly changed only in the model group are indicated by blue font. Black arrows indicate metabolic processes, whereas dashed arrows indicate omission of intermediates. Purple arrows indicate promotion, and the green T-shaped solid lines indicate inhibition. GR, glutathione reductase; GSH, glutathione.

To more clearly understand the relationship between the anomalous small molecules detected using MALDI–MSI, we identified ROS as important intermediaries in the abnormal changes of taurine, glutathione, ascorbic acid, aspartic acid, N-acetyl-aspartate, adenosine, and phospholipid levels (**Figure 10**); moreover, Shenfu injection effectively changed the taurine, glutathione, and phospholipid levels. In the past decade, the pharmaceutical industry has witnessed a shift from the pursuit of one active ingredient hitting a single target to seeking combination therapies that comprise several active ingredients, and given that Shenfu injection has been widely used in China for treating patients with septic shock, the present study suggests that Shenfu injection provides new treatment strategies of IHF in clinical settings.

## Data Availability Statement

The raw data supporting the conclusions of this manuscript will be made available by the authors, without undue resevation, to any qualified researcher.

## Ethics Statement

This study was carried out in accordance with the recommendations of Peking University Biomedical Ethics Committee (Beijing, China; approval no.: LA2017282). The protocol was approved by Peking University Biomedical Ethics Committee. The animal experimenters hold employment certificates from the Department of Laboratory Animal Science, Peking University Health Science Center.

## Author Contributions

HW designed and performed the experiments, analyzed and interpreted the data, and wrote the manuscript. ZD participated in performing the experiments. XL, ML, and ZG assisted in data analysis. FT participated in material support. XZ and YS revised the paper. XP designed the study and revised the manuscript. All authors read and approved the final manuscript.

## Funding

This study was supported by the following National Natural Science Foundation of China (Grant no.: U1603128), Science and Technology Major Projects: Significant New-Drugs Creation (Grant no.: 2018ZX09711001-009-006), and Sichuan Province Science and Technology Program (Grant no.: 2017JZ0036).

## Conflict of Interest

The authors declare that the research was conducted in the absence of any commercial or financial relationships that could be construed as a potential conflict of interest.

## Supplementary Material

Supplementary Table 1 Quality control of Shenfu injection.

Supplementary Figure 1 Flow chart of the Shenfu injection preparation process.

Supplementary Figure 2 Standardized analysis of Shenfu injection components using HPLC. **(A)** Six ginsenoside reference substances and **(B)** HPLC-fingerprint of Shenfu injection ginsenosides. **(C)** Three aconitine reference substances and **(D)** HPLC-fingerprint of Shenfu injection aconitines. **(E)** Chemical structure of the nine reference substances.

Supplementary Figure 3 Electrocardiogram of rats. **(A)** Normal electrocardiogram (50 mm/s; 10 mm/mV). **(B)** Myocardial ischemia electrocardiogram (50 mm/s; 10 mm/mV).

Supplementary Figure 4 Effect of Shenfu injection on the survival of the rats with ischemic heart failure. **(A)** Post-surgery to pre-therapy survival curve. **(B)** Post-therapy survival curve. N, normal; S, sham; All MOD and MOD, model; CAR, carvedilol; L, 3 ml/kg Shenfu injection; M, 6 ml/kg Shenfu injection; H, 12 ml/kg Shenfu injection. ^#^P < 0.05 vs. sham group.

Supplementary Figure 5 Effect of Shenfu injection on serum cardiac troponin T levels in the rats with ischemic heart failure. N, normal; S, sham; MOD, model; CAR, carvedilol; M, 6 ml/kg Shenfu injection; H, 12 ml/kg Shenfu injection. Data are expressed as mean ± standard error of the mean; n = 5 per group. ^##^P < 0.01 vs. S group; *P < 0.05 vs. MOD group.

Supplementary Figure 6 Effect of Shenfu injection on TCA cycle- and energy metabolism-related molecules in the hearts of the rats with ischemic heart failure. Intensity ratios of molecules were analyzed using the SCiLS Lab software in a normalized manner. The spatial resolution is 200 µm (Scale bar, 5 mm). S, sham; MOD, model; H, 12 ml/kg Shenfu injection. Data are presented as the mean ± standard error of the mean; n = 3 per group. ^#^P < 0.05, ^##^P < 0.01 vs. S group.

## References

[B1] Ammar M. R. Kassas N. Bader M. F. Vitale N. (2014). Phosphatidic acid in neuronal development: a node for membrane and cytoskeleton rearrangements. Biochimie 107 Pt A, 51–57. 10.1016/j.biochi.2014.07.026 25111738

[B2] Benjamin E. J. Virani S. S. Callaway C. W. Chamberlain A. M. Chang A. R. Cheng S. (2018). Heart disease and stroke statistics-2018 update: a report from the American Heart Association. Circulation. 137, e67–e492. 10.1161/CIR.0000000000000558 29386200

[B3] Chen W. W. Gao R. L. Liu L. S. Zhu M. L. Wang W. Wang Y. J. (2017). China cardiovascular diseases report 2015: a summary. J. Geriatr. Cardiol. 14, 1–10. 10.11909/j.issn.1671-5411.2017.01.012 28270835 PMC5329726

[B4] Dallak M. (2017). A synergistic protective effect of selenium and taurine against experimentally induced myocardial infarction in rats. Arch. Physiol. Biochem. 123, 344–355. 10.1080/13813455.2017.1347687 28699791

[B5] Freigang S. (2016). The regulation of inflammation by oxidized phospholipids. Eur. J. Immunol. 46, 1818–1825. 10.1002/eji.201545676 27312261

[B6] Grandinetti V. Carlos F. P. Antonio E. L. De-Oliveira H. A. Dos-Santos L. F. N. Yoshizaki A. (2019). Photobiomodulation therapy combined with carvedilol attenuates post-infarction heart failure by suppressing excessive inflammation and oxidative stress in rats. Sci. Rep. 9, 9425. 10.1038/s41598-019-46021-1 31263132 PMC6603025

[B7] Guo Z. J. Li C. S. (2013). Therapeutic effects of shenfu injection on post-cardiac arrest syndrome. Chin. J. Integr. Med. 19, 716–720. 10.1007/s11655-013-1566-8 23975138

[B8] Haidara M. A. Assiri A. S. Yassin H. Z. Ammar H. I. Obradovic M. M. Isenovic E. R. (2015). Heart failure models: traditional and novel therapy. Curr. Vasc. Pharmacol. 13, 658–669. 10.2174/1570161113666150212151506 25675330

[B9] Hjalmarson A. Waagstein F. (1994). The role of beta-blockers in the treatment of cardiomyopathy and ischaemic heart failure. Drugs. 47 Suppl 4, 31–39. 10.2165/00003495-199400474-00006 7523060

[B10] Liang A. H. Yi Y. Zhang Y. S. Han J. Y. Tian J. Z. Lu Y. T. (2015). Pseudoallergic reactions of traditional Chinese medicine injections and the approaches for risk prevention and control. Chin. Pharm. J. 15, 1301–1308.

[B11] Liang T. Zhang Y. Yin S. Gan T. An T. Zhang R. (2016). Cardio-protecteffect of qiliqiangxin capsule on left ventricular remodeling, dysfunction and apoptosis in heart failure rats after chronic myocardial infarction. Am. J. Transl. Res. 8, 2047–2058. 27347313 PMC4891418

[B12] Liu H. Chen R. Wang H. Chen S. Xiong C. Wang J. (2014). 1,5-Diaminonaphthalene hydrochloride assisted laser desorption/ionization mass spectrometry imaging of small molecules in tissues following focal cerebral ischemia. Anal. Chem. 86, 10114–10121. 10.1021/ac5034566 25247713

[B13] Liu H. Li W. He Q. Xue J. Wang J. Xiong C. (2017). Mass spectrometry imaging of kidney tissue sections of rat subjected to unilateral ureteral obstruction. Sci. Rep. 7, 41954. 10.1038/srep41954 28157191 PMC5291210

[B14] Liu X. Xu Y. Deng Y. Li H. (2018). MicroRNA-223 regulates cardiac fibrosis after myocardial infarction by targeting RASA1 . Cell. Physiol. Biochem. 46, 1439–1454. 10.1159/000489185 29689569

[B15] Lv F. H. Yin H. L. He Y. Q. Wu H. M. Kong J. Chai X. Y. (2016). Effects of curcumin on the apoptosis of cardiomyocytes and the expression of NF-kappaB, PPAR-gamma and Bcl-2 in rats with myocardial infarction injury. Exp. Ther. Med. 12, 3877–3884. 10.3892/etm.2016.3858 28105120 PMC5228430

[B16] Manickaraj S. Thirumalai D. Manjunath P. Sekarbabu V. Jeganathan S. Sundaresan L. (2017). Oxidative environment causes molecular remodeling in embryonic heart-a metabolomic and lipidomic fingerprinting analysis. Environ. Sci. Pollut. Res. Int. 24, 23825–23833. 10.1007/s11356-017-9997-y 28866837

[B17] Naghavi M. Abajobir A. A. Abbafati C. Abbas K. M. Abd-Allah F. Abera S. F. (2017). Global, regional, and national age-sex specific mortality for 264 causes of death, 1980-2016: a systematic analysis for the Global Burden of Disease Study 2016 . Lancet. 390, 1151–1210. 10.1016/S0140-6736(17)32152-9 28919116 PMC5605883

[B18] Nishizuka Y. (1992). Intracellular signaling by hydrolysis of phospholipids and activation of protein kinase C. Science. 258, 607–614. 10.1126/science.1411571 1411571

[B19] Pilla J. J. Koomalsingh K. J. Mcgarvey J. R. Witschey W. R. Dougherty L. Gorman J. H. (2015). Regional myocardial three-dimensional principal strains during postinfarction remodeling. Ann. Thorac. Surg. 99, 770–778. 10.1016/j.athoracsur.2014.10.067 25620591 PMC4352409

[B20] Shimada K. Jong C. J. Takahashi K. Schaffer S. W. (2015). Role of ROS production and turnover in the antioxidant activity of taurine. Adv. Exp. Med. Biol. 803, 581–596. 10.1007/978-3-319-15126-7_47 25833529

[B21] Smith S. A. Mammen P. P. Mitchell J. H. Garry M. G. (2003). Role of the exercise pressor reflex in rats with dilated cardiomyopathy. Circulation. 108, 1126–1132. 10.1161/01.CIR.0000084538.40542.56 12925464

[B22] Smith S. A. Williams M. A. Mitchell J. H. Mammen P. P. Garry M. G. (2005). The capsaicin-sensitive afferent neuron in skeletal muscle is abnormal in heart failure. Circulation. 111, 2056–2065. 10.1161/01.CIR.0000162473.10951.0A 15851614

[B23] Stathopoulou K. Wittig I. Heidler J. Piasecki A. Richter F. Diering S. (2016). S-glutathiolation impairs phosphoregulation and function of cardiac myosin-binding protein C in human heart failure. Faseb. J. 30, 1849–1864. 10.1096/fj.201500048 26839380 PMC4836368

[B24] Takagawa J. Zhang Y. Wong M. L. Sievers R. E. Kapasi N. K. Wang Y. (2007). Myocardial infarct size measurement in the mouse chronic infarction model: comparison of area- and length-based approaches. J. Appl. Physiol. 102, 2104–2111. 10.1152/japplphysiol.00033.2007 17347379 PMC2675697

[B25] Takemura G. Kanamori H. Okada H. Miyazaki N. Watanabe T. Tsujimoto A. (2018). Anti-apoptosis in nonmyocytes and pro-autophagy in cardiomyocytes: two strategies against postinfarction heart failure through regulation of cell death/degeneration. Heart. Fail. Rev. 23, 759–772. 10.1007/s10741-018-9708-x 29737434

[B26] Talameh J. A. Lanfear D. E. (2012). Pharmacogenetics in chronic heart failure: new developments and current challenges. Curr. Heart. Fail. Rep. 9, 23–32. 10.1007/s11897-011-0076-2 22135185 PMC3917307

[B27] Tang H. Y. Wang C. H. Ho H. Y. Wu P. T. Hung C. L. Huang C. Y. (2018). Lipidomics reveals accumulation of the oxidized cholesterol in erythrocytes of heart failure patients. Redox Biol. 14, 499–508. 10.1016/j.redox.2017.10.020 29101899 PMC5675899

[B28] Tappia P. S. Yu C. H. Di Nardo P. Pasricha A. K. Dhalla N. S. Panagia V. (2001). Depressed responsiveness of phospholipase C isoenzymes to phosphatidic acid in congestive heart failure. J. Mol. Cell. Cardiol. 33, 431–440. 10.1006/jmcc.2000.1315 11181012

[B29] Wang Y. L. Wang C. Y. Zhang B. J. Zhang Z. Z. (2009). Shenfu injection suppresses apoptosis by regulation of Bcl-2 and caspase-3 during hypoxia/reoxygenation in neonatal rat cardiomyocytes in vitro. Mol. Biol. Rep. 36, 365–370. 10.1007/s11033-007-9188-x 18049909

[B30] Wang X. Guo Z. Ding Z. Mehta J. L. (2018). Inflammation, autophagy, and apoptosis after myocardial infarction. J. Am. Heart. Assoc. 7, e008024. 10.1161/JAHA.117.008024 29680826 PMC6015297

[B31] Wei P. Yang X. J. Fu Q. Han B. Ling L. Bai J. (2015). Intermedin attenuates myocardial infarction through activation of autophagy in a rat model of ischemic heart failure via both cAMP and MAPK/ERK1/2 pathways. Int. J. Clin. Exp. Pathol. 8, 9836–9844. 26617693 PMC4637778

[B32] Wu Y. Xia Z. Y. Meng Q. T. Zhu J. Lei S. Xu J. (2011). Shen-Fu injection preconditioning inhibits myocardial ischemia-reperfusion injury in diabetic rats: activation of eNOS via the PI3K/Akt pathway. J. Biomed. Biotechnol. 2011, 384627. 10.1155/2011/384627 21151615 PMC2997576

[B33] Xing J. Lu J. Li J. (2014). Nerve growth factor decreases in sympathetic and sensory nerves of rats with chronic heart failure. Neurochem. Res. 39, 1564–1570. 10.1007/s11064-014-1348-5 24913185 PMC4125521

[B34] Yancy C. W. Jessup M. Bozkurt B. Butler J. Casey D.E. Jr. Drazner M. H. (2013). 2013 ACCF/AHA guideline for the management of heart failure: a report of the American College of Cardiology Foundation/American Heart Association Task Force on Practice Guidelines. J. Am. Coll. Cardiol. 62, e147–e239. 23747642 10.1016/j.jacc.2013.05.019

[B35] Zheng S. Wu H. Yu S. Ren J. Duo W. Ma Z. (2015). Shenfu Injection suppresses inflammation by targeting haptoglobin and pentraxin 3 in rats with chronic ischemic heart failure. Chin. J. Integr. Med. 21, 22–28. 10.1007/s11655-013-1440-8 23494325

