# Supplementary material for: Pharmacodynamic Evaluation of Shenfu Injection in Rats With Ischemic Heart Failure and Its Effect on Small Molecules Using Matrix-Assisted Laser Desorption/Ionization–Mass Spectrometry Imaging
Source: Front Pharmacol. 2019 Nov 26;10:1424. doi: 10.3389/fphar.2019.01424 (PMC6889858; doi:10.3389/fphar.2019.01424)
Supplement: Supplementary Table 1 — Quality control of Shenfu injection. [file Table_1.docx]

Supplementary Material

# Supplementary Table

**Supplementary Table 1.** Quality control of Shenfu injection.

| **Compound** | **Concentration** | **Standard regulatory concentration** |
| --- | --- | --- |
| Ginsenoside Rg1  Ginsenoside Re  Ginsenoside Rb1  Benzoylmesaconine | 0.13 mg/ml  0.12 mg/ml  1.4 mg/ml  1.60 μg/ml | >0.04 mg/ml  >0.02 mg/ml  0.6–1.8 mg/ml  0.50–4.50 μg/ml |
