# Supplementary figures and images for: Pharmacodynamic Evaluation of Shenfu Injection in Rats With Ischemic Heart Failure and Its Effect on Small Molecules Using Matrix-Assisted Laser Desorption/Ionization–Mass Spectrometry Imaging
Source: Front Pharmacol. 2019 Nov 26;10:1424. doi: 10.3389/fphar.2019.01424 (PMC6889858; doi:10.3389/fphar.2019.01424)

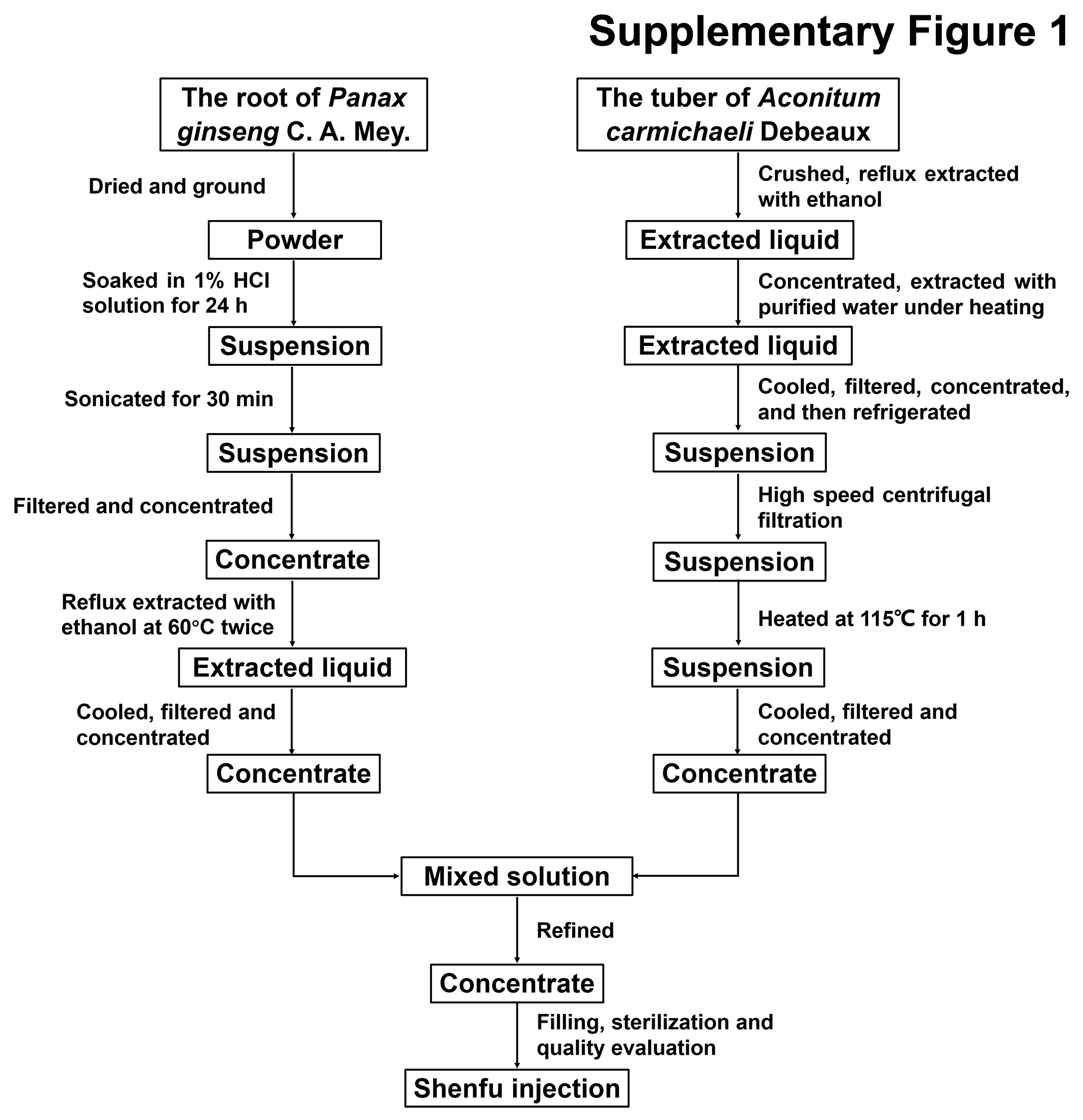

Supplement: Supplementary Figure 1 — Flow chart of the Shenfu injection preparation process. [file Image_1.tif]

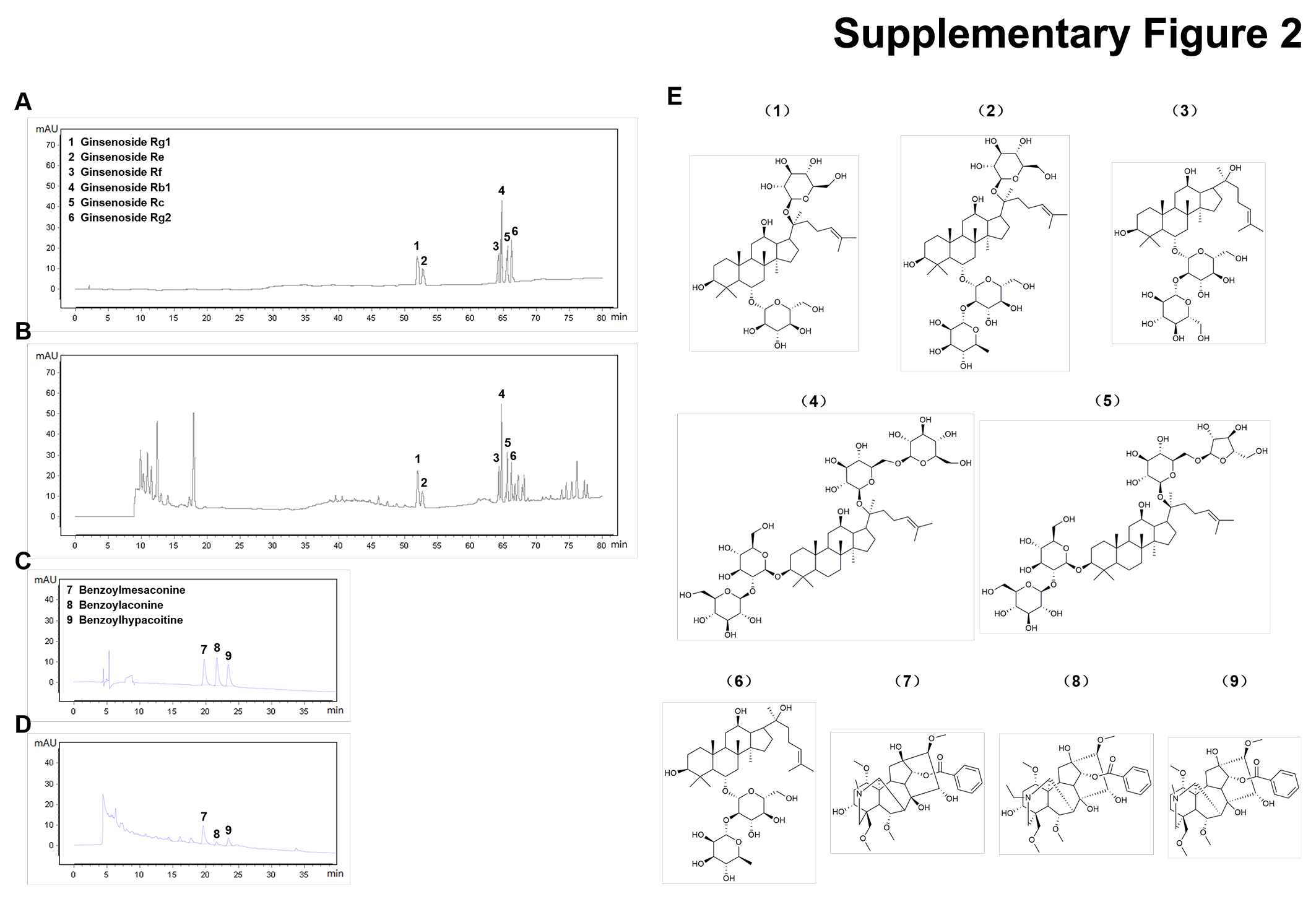

Supplement: Supplementary Figure 2 — Standardized analysis of Shenfu injection components using HPLC. (A) Six ginsenoside reference substances and (B) HPLC-fingerprint of Shenfu injection ginsenosides. (C) Three aconitine reference substances and (D) HPLC-fingerprint of Shenfu injection aconitines. (E) Chemical structure of the nine reference substances. [file Image_2.tif]

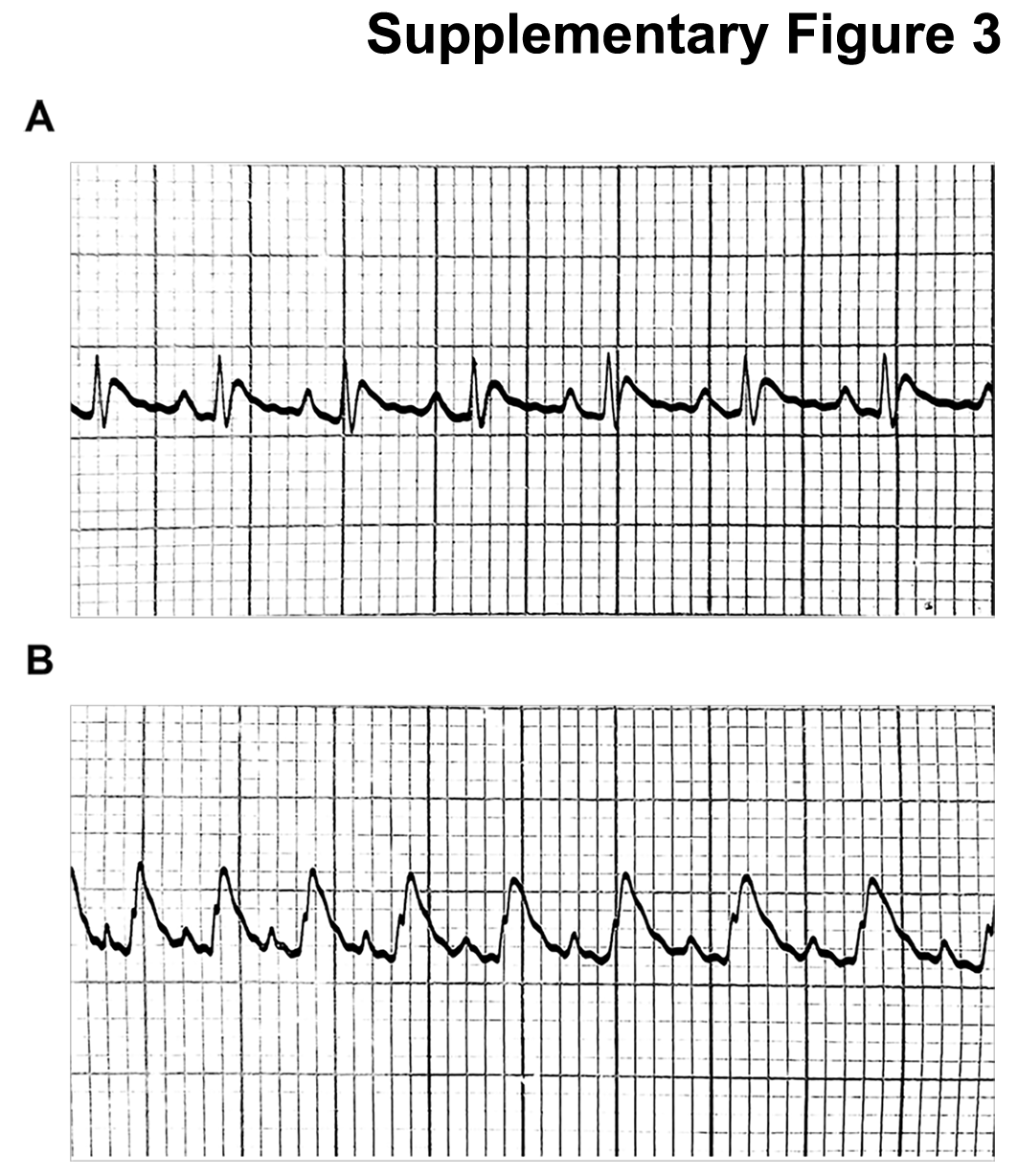

Supplement: Supplementary Figure 3 — Electrocardiogram of rats. (A) Normal electrocardiogram (50 mm/s; 10 mm/mV). (B) Myocardial ischemia electrocardiogram (50 mm/s; 10 mm/mV). [file Image_3.tif]

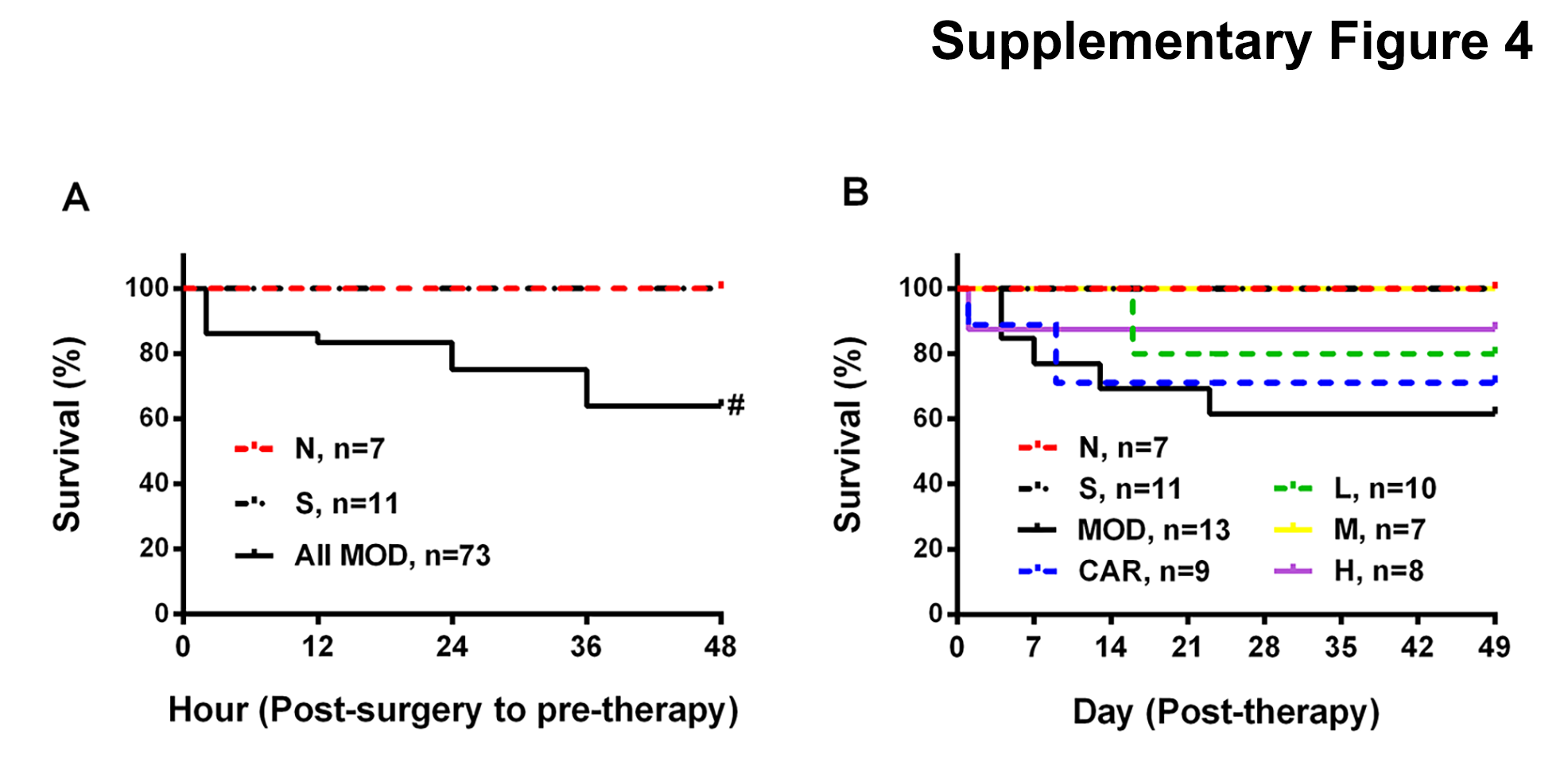

Supplement: Supplementary Figure 4 — Effect of Shenfu injection on the survival of the rats with ischemic heart failure. (A) Post-surgery to pre-therapy survival curve. (B) Post-therapy survival curve. N, normal; S, sham; All MOD and MOD, model; CAR, carvedilol; L, 3 ml/kg Shenfu injection; M, 6 ml/kg Shenfu injection; H, 12 ml/kg Shenfu injection. #P < 0.05 vs. sham group. [file Image_4.tif]

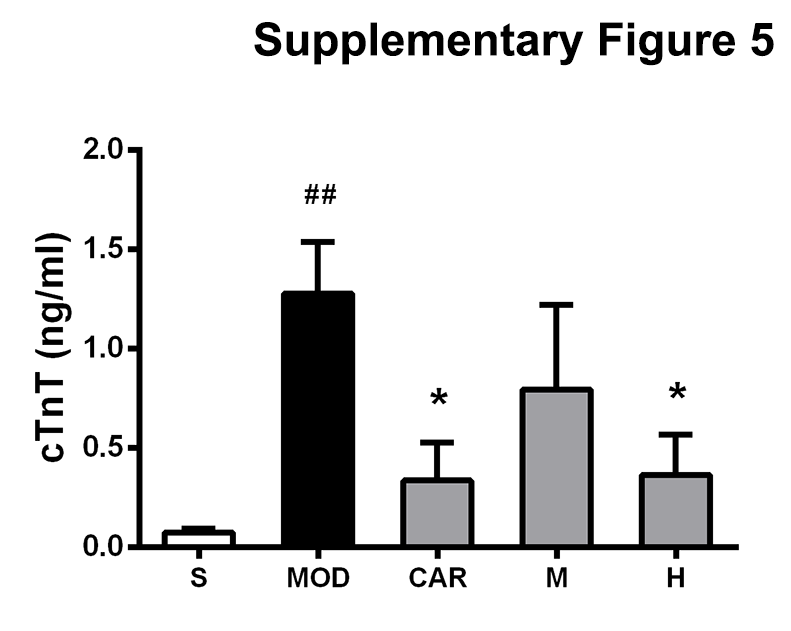

Supplement: Supplementary Figure 5 — Effect of Shenfu injection on serum cardiac troponin T levels in the rats with ischemic heart failure. N, normal; S, sham; MOD, model; CAR, carvedilol; M, 6 ml/kg Shenfu injection; H, 12 ml/kg Shenfu injection. Data are expressed as mean ± standard error of the mean; n = 5 per group. ##P < 0.01 vs. S group; *P < 0.05 vs. MOD group. [file Image_5.tif]

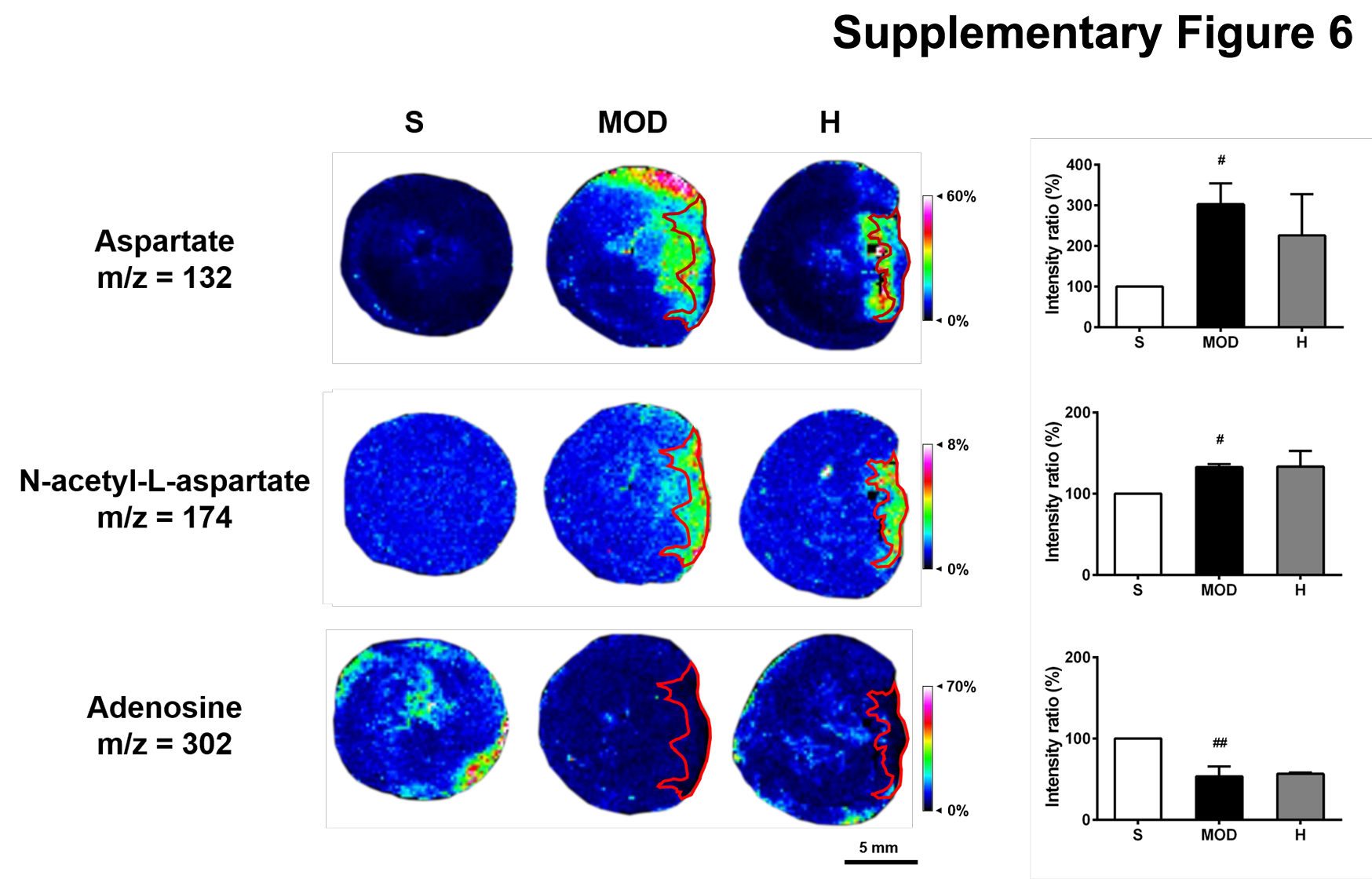

Supplement: Supplementary Figure 6 — Effect of Shenfu injection on TCA cycle- and energy metabolism-related molecules in the hearts of the rats with ischemic heart failure. Intensity ratios of molecules were analyzed using the SCiLS Lab software in a normalized manner. The spatial resolution is 200 µm (Scale bar, 5 mm). S, sham; MOD, model; H, 12 ml/kg Shenfu injection. Data are presented as the mean ± standard error of the mean; n = 3 per group. #P < 0.05, ##P < 0.01 vs. S group. [file Image_6.tif]
